# Treatment with a β-2-adrenoceptor agonist stimulates glucose uptake in skeletal muscle and improves glucose homeostasis, insulin resistance and hepatic steatosis in mice with diet-induced obesity

**DOI:** 10.1007/s00125-020-05171-y

**Published:** 2020-05-29

**Authors:** Anastasia Kalinovich, Nodi Dehvari, Alice Åslund, Sten van Beek, Carina Halleskog, Jessica Olsen, Elisabete Forsberg, Evelyn Zacharewicz, Gert Schaart, Mia Rinde, Anna Sandström, Roger Berlin, Claes-Göran Östenson, Joris Hoeks, Tore Bengtsson

**Affiliations:** 1 Department of Molecular Biosciences, the Wenner-Gren Institute, Stockholm University, Svante Arrhenius väg 20B, Arrhenius laboratories F3, 10691 Stockholm, Sweden; 2 Atrogi AB, Stockholm, Sweden; 3 Department of Nutrition and Movement Sciences, Maastricht University, Maastricht, the Netherlands; 4 Present Address: Takeda AB, Stockholm, Sweden; 5 Department of Molecular Medicine and Surgery, Endocrine and Diabetes Unit, Karolinska Institute, Karolinska University Hospital, Stockholm, Sweden

**Keywords:** β_2_-Adrenergic signalling, Clenbuterol, Hepatic steatosis, Insulin resistance, Skeletal muscle, Type 2 diabetes

## Abstract

**Aims/hypothesis:**

Chronic stimulation of β_2_-adrenoceptors, opposite to acute treatment, was reported to reduce blood glucose levels, as well as to improve glucose and insulin tolerance in rodent models of diabetes by essentially unknown mechanisms. We recently described a novel pathway that mediates glucose uptake in skeletal muscle cells via stimulation of β_2_-adrenoceptors. In the current study we further explored the potential therapeutic relevance of β_2_-adrenoceptor stimulation to improve glucose homeostasis and the mechanisms responsible for the effect.

**Methods:**

C57Bl/6N mice with diet-induced obesity were treated both acutely and for up to 42 days with a wide range of clenbuterol dosages and treatment durations. Glucose homeostasis was assessed by glucose tolerance test. We also measured in vivo glucose uptake in skeletal muscle, insulin sensitivity by insulin tolerance test, plasma insulin levels, hepatic lipids and glycogen.

**Results:**

Consistent with previous findings, acute clenbuterol administration increased blood glucose and insulin levels. However, already after 4 days of treatment, beneficial effects of clenbuterol were manifested in glucose homeostasis (32% improvement of glucose tolerance after 4 days of treatment, *p* < 0.01) and these effects persisted up to 42 days of treatment. These favourable metabolic effects could be achieved with doses as low as 0.025 mg kg^−1^ day^−1^ (40 times lower than previously studied). Mechanistically, these effects were not due to increased insulin levels, but clenbuterol enhanced glucose uptake in skeletal muscle in vivo both acutely in lean mice (by 64%, *p* < 0.001) as well as during chronic treatment in diet-induced obese mice (by 74%, *p* < 0.001). Notably, prolonged treatment with low-dose clenbuterol improved whole-body insulin sensitivity (glucose disposal rate after insulin injection increased up to 1.38 ± 0.31%/min in comparison with 0.15 ± 0.36%/min in control mice, *p* < 0.05) and drastically reduced hepatic steatosis (by 40%, *p* < 0.01) and glycogen (by 23%, *p* < 0.05).

**Conclusions/interpretation:**

Clenbuterol improved glucose tolerance after 4 days of treatment and these effects were maintained for up to 42 days. Effects were achieved with doses in a clinically relevant microgram range. Mechanistically, prolonged treatment with a low dose of clenbuterol improved glucose homeostasis in insulin resistant mice, most likely by stimulating glucose uptake in skeletal muscle and improving whole-body insulin sensitivity as well as by reducing hepatic lipids and glycogen. We conclude that selective β_2_-adrenergic agonists might be an attractive potential treatment for type 2 diabetes. This remains to be confirmed in humans.

**Figure:**
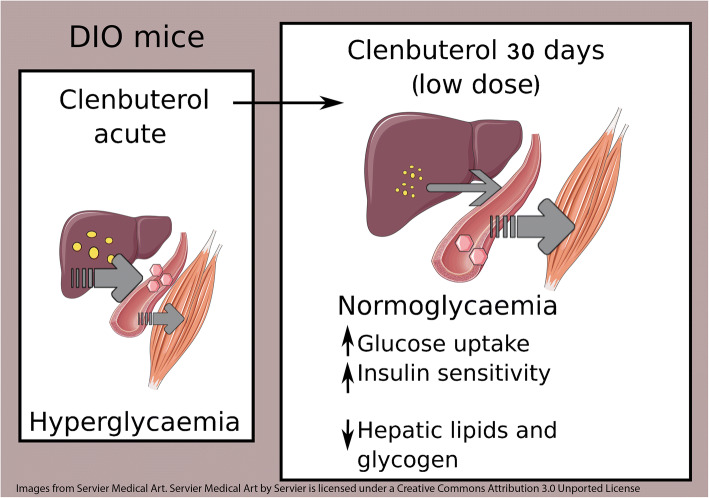
Graphical abstract

**Electronic supplementary material:**

The online version of this article (10.1007/s00125-020-05171-y) contains peer-reviewed but unedited supplementary material, which is available to authorised users.

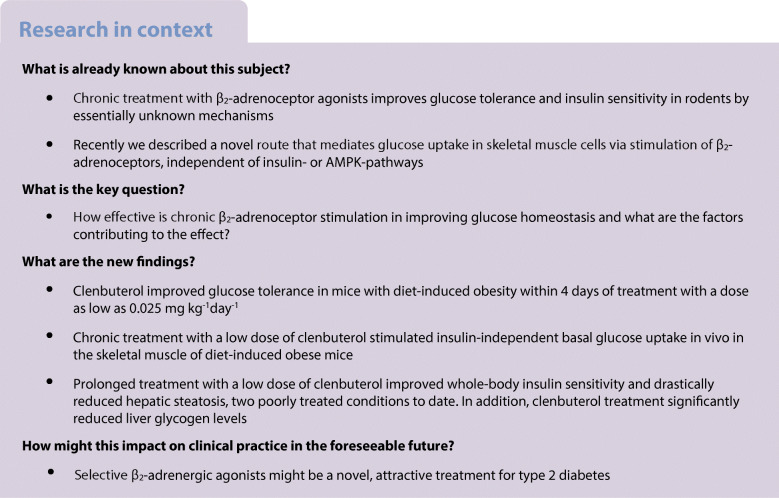



## Introduction

Type 2 diabetes mellitus is characterised by impaired glucose homeostasis resulting from insulin resistance in peripheral tissues, such as liver and skeletal muscle, as well as from relative impairments in insulin release by pancreatic β-cells. Skeletal muscle is responsible for up to 80% of insulin-stimulated glucose uptake in healthy individuals [1], but glucose uptake in skeletal muscle of diabetic patients is severely impaired owing to insulin resistance. It makes it an attractive target to improve glucose homeostasis. However, there are currently no approved glucose-lowering drugs targeting glucose uptake in skeletal muscles.

We have recently described an entirely novel pathway that stimulates GLUT4-mediated glucose uptake in skeletal muscle cells. This pathway operates independent of the activation of classical regulatory pathways (insulin signalling or AMP-activated protein kinase [AMPK]) but instead involves β_2_-adrenergic receptors (β_2_-AR) and the activation of the mammalian target of rapamycin complex 2 (mTORC2) [2]. We demonstrated that β_2_-AR stimulation induced glucose uptake both in vitro in myotubes and in vivo in skeletal muscle of healthy mice (after acute single injection with a relatively high dose of adrenergic agonists) [2, 3]. In addition, we and others have also shown that β_2_-AR stimulation, using a relatively high dosage of the β_2_-AR agonist clenbuterol, improved glucose tolerance in diet-induced obese (DIO) mice [2], Goto–Kakizaki rats [2] and Zucker fatty rats [4]—well-established rodent models for human type 2 diabetes. In line with these data, β_2_-AR-ablation resulted in hyperglycaemia in mice [5, 6].

The beneficial effects described above are not obvious, since it is an accepted fact that β_2_-AR agonists administered acutely cause hyperglycaemia (due to enhanced glucose output from the liver) and hyperinsulinaemia in humans and rodents [7–10] and have even been proposed for treatment of hypoglycaemic incidents [11, 12]. The mechanism(s) explaining the beneficial effects of prolonged treatment vs deleterious acute effects with β_2_-AR agonists in vivo have been discussed [13, 14], but remain undefined. Thus, we find it important in the present study to highlight and discuss further the difference between acute and chronic treatment with β-AR agonists.

Besides the beneficial effects on glucose tolerance, chronic treatment with clenbuterol or other β-AR agonists improves insulin resistance [13–19]. The underlying mechanism by which β-adrenergic agonists beneficially affect insulin sensitivity remains to be elucidated. In the present study we discuss possible contributing factors. While potentiation of insulin-stimulated glucose uptake in muscle was reported for several β-AR agonists, including clenbuterol [15, 19], basal glucose disposal has not been sufficiently studied, despite its major role in maintaining fasting blood glucose. For this purpose, we studied the effect of β_2_-AR agonists on basal glucose uptake (in the fasting state with low insulin levels) during chronic treatment.

Taken together, the current work aimed to further explore the therapeutic relevance of β_2_-AR stimulation on glucose homeostasis. To develop our understanding of this matter, we chose clenbuterol as an oral long-lasting selective β_2_-AR agonist, since it has been studied most extensively in the field and particularly it has been demonstrated to stimulate glucose uptake in myocytes in vitro [2]. Clenbuterol has an unfavourable reputation for its misuse in high doses by bodybuilders and fitness enthusiasts because of its hypertrophic effects in muscles and lipolytic effects in adipose tissues. However, clenbuterol in lower doses is generally assumed to be safe [20–22] and is used in humans for asthma treatment in a number of countries. The current study was performed on a well-established model of type 2 diabetes, DIO mice, which are characterised by obesity, glucose intolerance, insulin resistance and hepatic steatosis. Moreover, DIO mice share the same origin of diabetes with the majority of diabetic patients—obesity-related.

In the current study we first treated DIO mice both acutely and chronically with a wide range of clenbuterol dosages to determine the minimal dose needed to improve glucose tolerance, which has not been studied before. Second, we discussed the mechanistical difference between acute and chronic treatment with β_2_-AR agonists. And finally, to explore the mechanisms involved, we treated DIO mice chronically with a relatively low dose of clenbuterol (in comparison with previously used dosages [2, 4, 15]) and performed ITT, and measured plasma insulin levels, basal glucose uptake in skeletal muscle as well as hepatic lipids and glycogen.

## Methods

For detailed methods, please refer to the electronic supplementary material (ESM).

### Animals

C57Bl/6N mice were bred at Stockholm University or purchased from Scanbur (Charles River). To generate diet-induced obesity, mice were fed a high-fat (45% fat) diet (HFD) (D12451, Research Diets) ad libitum for 4–9 months and kept at thermoneutrality (30°C) with a 12 h light/dark cycle and unlimited access to water. Mean body weight of DIO mice was 40–50 g. DIO mice were males unless stated otherwise and 7–10 months old at the beginning of the treatment. Routinely, mice were housed in individual cages during the treatment. Cages were enriched with wood chips, a cardboard house or a roll, a wooden stick, paper and a piece of cotton. Body composition was measured by EchoMRI-100 (Echo Medical Systems). All procedures were approved by the North Stockholm Ethical Committee for Care and Use of Laboratory Animals.

### Treatment

Prior to treatment mice in different experimental groups had similar glucose tolerance, body weight and body composition. Clenbuterol (C5423, Sigma Aldrich) was injected i.p. daily (dissolved in saline [154 mmol/l NaCl]) or supplied in drinking water. We defined acute treatment as administration of a single dose and chronic (or prolonged) treatment as multiple dose treatment for 4 days or longer. Mice were treated in random order.

### GTT, ITT and pyruvate tolerance test

Clenbuterol was not administered on the day of a test. Mice were fasted for 5 h (prior to GTT and ITT) or 12 h (prior to pyruvate tolerance test). For GTT, glucose 2.5 g/kg lean weight (unless stated otherwise) was either injected i.p. or orally gavaged. For ITT and pyruvate tolerance test, human insulin 1 U/kg body weight (Actrapid, NovoNordisk) or pyruvate 2.5 g/kg lean weight (P8574, Sigma Aldrich) was injected i.p. In OGTTs, IPGTTs and pyruvate tolerance test, the total AUC was calculated with 0 mmol/l blood glucose as a baseline. Please note that the effect on fasting blood glucose contributes to the effect on total AUC. During OGTTs, plasma insulin was measured at time points 0 and 15 min by Elisa kit (90080, CrystalChem). In ITT, the rate of blood glucose disposal (*K*_ITT_) was calculated as 0.693 × 100/t_1/2_, where t_1/2_ is the time necessary to reduce blood glucose by half, calculated from the linear regression analysis of data at 0, 15 and 30 min [23]. Mice were accessed in random order.

### In vivo glucose uptake

**Fig. 1:**
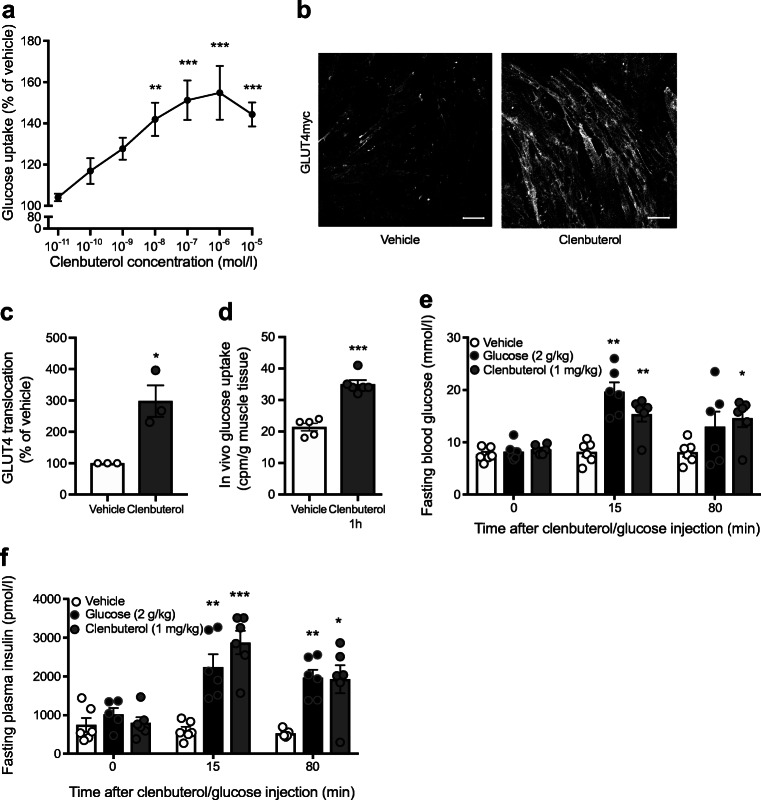
In vitro and acute in vivo effects of clenbuterol. (**a**) Clenbuterol stimulation of L6 myotubes induced glucose uptake in a dose-dependent manner; *n* = 5–8; data were analysed by one-way ANOVA with Dunnett’s multiple comparison test. (**b**, **c**) Stimulation of L6 myotubes stably expressing GLUT4myc with 1 μmol/l clenbuterol induces GLUT4 translocation as quantified in (**c**); *n* = 3; data were analysed with unpaired two-tailed Student’s *t* test. Scale bars, 50 μm. (**d**) In vivo glucose uptake in gastrocnemius muscle of chow-fed mice treated with 1 mg/kg clenbuterol for 1 h; *n* = 5–6; data were analysed with unpaired two-tailed Student’s *t* test. (**e**, **f**) Acute effects of clenbuterol on blood glucose (**e**) and plasma insulin (**f**); *n* = 6 (diet-induced obesity was developed in C57Bl/6N mice maintained at 30°C and on HFD for 7 months; mice were fasted in the morning for 6 h prior to clenbuterol, glucose or saline i.p. injection); data were analysed by two-way ANOVA with Dunnett’s multiple comparison test. In all graphs: *p < 0.05, **p < 0.01, ***p < 0.001 vs vehicle (in e and f vs vehicle at the same time point)

Mice were fasted for 5 h, anaesthetised with pentobarbital (60 mg/kg i.p.), injected with clenbuterol (1 mg/kg, i.p.) or saline, and 20 min later injected i.p. with 4.81 × 10^6^ Bq/kg of 2-deoxy[^3^H]glucose (Perkin Elmer, Waltham, MA, USA; 2.96 × 10^11^ Bq/mmol) and euthanised 1 h later (as shown in Fig. 1d).

Mice were fasted for 5 h, and 5 h after the last treatment with saline/clenbuterol, mice were anaesthetised with pentobarbital (70 mg/kg i.p.), injected i.p. with 4.81 × 10^6^ Bq/kg of 2-deoxy[^3^H]glucose and 80 min later euthanised. Serum was collected 20 min after 2-deoxy[^3^H]glucose injection, and insulin was measured as described above (as shown in Fig. 7a–b).

Gastrocnemius muscle was lysed in NaOH and analysed in a beta-counter. Mice were accessed in mixed order and investigators were blinded to treatment.

### Hepatic lipids

Fluorescence dye BODIPY 495/503 (0.1 mg/ml; D3922, Molecular Probes) was applied to frozen liver sections for 90 min at 37°C. Images were obtained using E800-fluorescence microscope (Nikon).

Total lipids were extracted by two sequential incubations with 5 ml and 2 ml of methanol:chloroform extraction medium (1:2, vol./vol.) at room temperature for 2 days each time. Extraction media with lipids was collected and evaporated and lipid weight was determined gravimetrically.

### Hepatic glycogen

A piece of liver was homogenised in water, boiled, centrifuged for 10 min at 18,000 *g* and the supernatant was assayed using a kit (ab65620, Abcam).

### Cell cultures

L6 rat myoblasts and L6 myoblasts stably expressing GLUT4myc were purchased from KeraFast (ESK201 and ESK202), where they were tested for mycoplasma. Normal morphology and growth were always controlled. Cells were grown 90% to confluence and differentiated until formation of myotubes (5–7 days).

### In vitro glucose uptake

Differentiated L6 cells were serum-starved for 3.5 h, stimulated for 1.5 h with clenbuterol, washed with glucose-free media, stimulated with clenbuterol/saline for another 20 min, exposed to 50 nmol/l 2-deoxy[^3^H]glucose (Perkin Elmer, Waltham MA USA; 2.96 × 10^11^ Bq/mmol) for 10 min, washed in glucose-free medium, lysed with NaOH, mixed with scintillation buffer and assayed in a beta-counter.

### In vitro GLUT4 translocation

Differentiated L6 cells stably expressing GLUT4-myc were serum-starved for 3 h, stimulated for 2 h with 1 μmol/l clenbuterol or vehicle, fixed with 2% paraformaldehyde, quenched with glycine and blocked with BSA, incubated with primary antibody (rabbit anti-myc, 2278 from Cell Signaling, diluted 1:500 in PBS with 5% BSA) overnight in 4°C, incubated in the dark for 1 h with conjugated Alexa Fluor555 goat anti-rabbit antibody (21429 from Invitrogen, diluted 1:500 in PBS with 1.5% BSA). Fluorescence was detected with a fluorescent confocal microscope (Zeiss LSM 800). When myc-epitope was probed on the cells by Western blot, it resulted in only one band of a right molecular weight (not shown). Omission of the primary antibody resulted in no staining of the cells, confirming specificity of the secondary antibody.

### Statistical analysis

Data are expressed as the mean ± SEM. Each data point is a single mouse or, in cell experiments, a mean of duplicates or triplicates from separate experiments. Criteria for data exclusions were: obvious pippeting errors using insulin ELISA kits, which resulted in almost no signal (one value from each of the control and glucose groups in Fig. 1f; one control value on Fig. 5a and one treated value on Fig. 5b); improperly injected glucose during IPGTT that did not result in a rise in blood glucose (two control values in Fig. 3a, b); water leakage resulted in too high apparent water intake (few days in all groups in Fig. 4a). Data were analysed with unpaired two-tailed Student’s *t* test, or one-way or two-way ANOVA with the Dunnett’s or Sidak’s multiple comparison tests as indicated in figure legends. Statistical analyses were performed using GraphPad Prism 8.2. A significant difference was considered at * *p* < 0.05, ** *p* < 0.01, *** *p* < 0.001.

## Results

### Beneficial effects of clenbuterol on glucose tolerance occur after 4 days of treatment and persist for a prolonged period of time

Previously, we have shown significant increases in glucose uptake in L6 myotubes upon acute incubation with the β_2_-adrenergic agonist clenbuterol [2]. To confirm and elaborate on these results, we demonstrated that acute clenbuterol-mediated glucose uptake in L6 cells occurs in a dose-dependent manner, with doses as low as 10^−8^ mol/l significantly enhancing glucose uptake by 42% (*p* < 0.01, Fig. 1a). We also showed that clenbuterol stimulation significantly enhanced GLUT4 translocation in L6 myotubes (by 298%, *p* < 0.05, Fig. 1b, c). Moreover, clenbuterol stimulated glucose uptake in vivo in skeletal muscle when administered acutely in healthy mice (64%, *p* < 0.001, Fig. 1d).

**Fig. 2:**
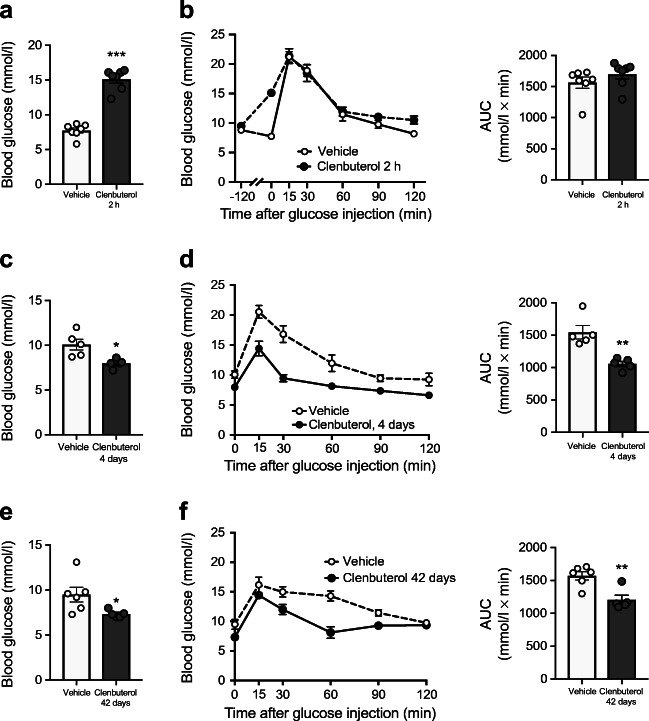
Prolonged clenbuterol treatment improves glucose tolerance in DIO mice. (**a**, **b**) Effects of acute 2 h pre-treatment with clenbuterol on fasting blood glucose (**a**) and IPGTT (**b**) in DIO mice. Diet-induced obesity was developed in C57Bl/6N mice maintained at 30°C and on HFD for 6 months; mice were fasted for 5 h, clenbuterol (1 mg/kg) was injected i.p. at −120 min; glucose (2 g/kg body weight) was injected i.p. at 0 min after blood glucose measurement; *n* = 7. (**c**, **d**) Chronic 4 day treatment with clenbuterol decreased fasting blood glucose (**c**) and improved i.p. glucose tolerance (**d**). Diet-induced obesity was developed in C57Bl/6N mice maintained at 30°C and on HFD for 7 months; clenbuterol (1 mg/kg) was injected i.p. every morning for 4 days; on the 5th day mice were fasted for 5 h, glucose (2 g/kg body weight) was injected i.p.; *n* = 5. (**e**, **f**) Chronic 42 day treatment with clenbuterol decreased fasting blood glucose (**e**) and improved i.p. glucose tolerance (**f**). Diet-induced obesity was developed in C57Bl/6N mice maintained at 30°C and on HFD for 7 months; clenbuterol (30 mg/l) was administered in drinking water; mean dose received was 2.04 ± 0.1 mg kg^−1^ day^−1^; mice were fasted for 5 h, glucose (2 g/kg body weight) was injected i.p.; *n* = 5–6. AUCs and fasting blood glucose were analysed with unpaired two-tailed Student’s *t* test. *p < 0.05, **p < 0.01, ***p < 0.001 vs vehicle

Next, we examined effects of acute clenbuterol administration on whole-body glucose homeostasis in vivo. Acute i.p. injection of clenbuterol (1 mg/kg) in DIO mice increased fasting blood glucose and insulin levels compared with a saline injection, up to similar levels to those observed during IPGTT (Fig. 1e, f). Glucose tolerance was not affected by acute clenbuterol treatment when analysed as total AUC (Fig. 2a, b); however, it was improved if we take into account the higher initial glucose levels (AUC above fasting blood glucose, not shown). Nonetheless, it should be considered that stimulated insulin release could contribute to this acute effect.

Interestingly, and in line with our previous study [2], when the clenbuterol treatment was continued for 4 days, we observed a reduction in fasting blood glucose (by 21%, *p* < 0.05) as well as an improved glucose tolerance (by 32%, *p* < 0.01, Fig. 2c, d, 24 h after last drug administration). Please note that the effect on fasting blood glucose contributes to the effect on total AUC. Surprisingly, repetitive daily drug administration did not increase blood glucose even transiently during chronic treatment (ESM Fig. 1). These beneficial effects of clenbuterol persisted for up to 42 days of treatment (Fig. 2e, f). It is important to note here that the beneficial effects of chronic treatment were not attributed to elevated insulin levels, in contrast to the acute effects (see below).

### Chronic treatment with clenbuterol improves glucose tolerance at doses in the microgram range

**Fig. 3:**
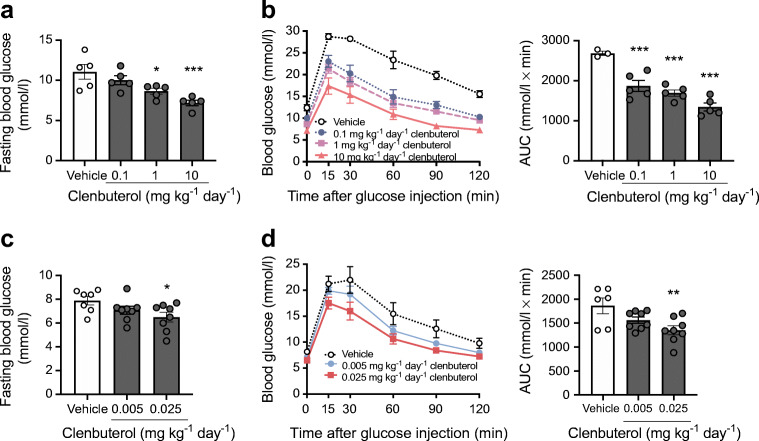
Clenbuterol improves fasting blood glucose and glucose tolerance in a dose-dependent manner. Two experiments were performed. In one experiment (**a**, **b**) DIO mice were treated with 0.1, 1 or 10 mg/kg of clenbuterol for 4 days; *n* = 3–5. Diet-induced obesity was developed in C57Bl/6N mice maintained at 30°C and on HFD for 9 months; clenbuterol was injected i.p. for 4 days; mice were fasted for 5 h, glucose (2 g/kg body weight) was injected i.p.; (**a**) fasting blood glucose and (**b**) glucose tolerance. In a separate experiment (**c**, **d**) DIO mice were treated with 0.005 or 0.025 mg/kg of clenbuterol for 4 days; *n* = 6–8, both males and females were used. Diet-induced obesity was developed in C57Bl/6N mice maintained at 30°C and on HFD for 4.5 months; clenbuterol was injected i.p. for 4 days; mice were fasted for 5 h, glucose (2 g/kg body weight) was injected i.p.; (**c**) fasting blood glucose and (**d**) glucose tolerance. AUCs and fasting blood glucose were analysed with one-way ANOVA with Dunnett’s multiple comparison test. In all graphs: *p < 0.05, **p < 0.01, ***p < 0.001 vs vehicle

In a dose–response study, DIO mice were treated daily with different doses of clenbuterol for 4 days, as this duration was shown to be sufficiently long to reach full effects. As seen in Fig. 3a, b, clenbuterol improved glucose tolerance and reduced fasting glucose levels in a dose-dependent manner. A subsequent experiment in a second cohort of mice indicated that a dose as low as 25 μg kg^−1^ day^−1^ (0.025 mg kg^−1^ day^−1^) still improved glucose tolerance (27%, *p* < 0.01) and reduced fasting blood glucose levels (18%, *p* < 0.05) (Fig. 3c, d). Taken together, clenbuterol improves glucose tolerance in DIO mice already after 4 days of treatment with doses in the microgram range.

### Chronic treatment with low-dose clenbuterol does not affect insulin release but improves insulin sensitivity

Since clenbuterol acutely leads to insulin release (Fig. 1f), the positive effects of prolonged clenbuterol treatment on glucose homeostasis could be, in part, due to changes in insulin secretion. To address this question, DIO mice were treated for 32 days with low-dose clenbuterol-supplemented water (3 mg/l).

**Fig. 4:**
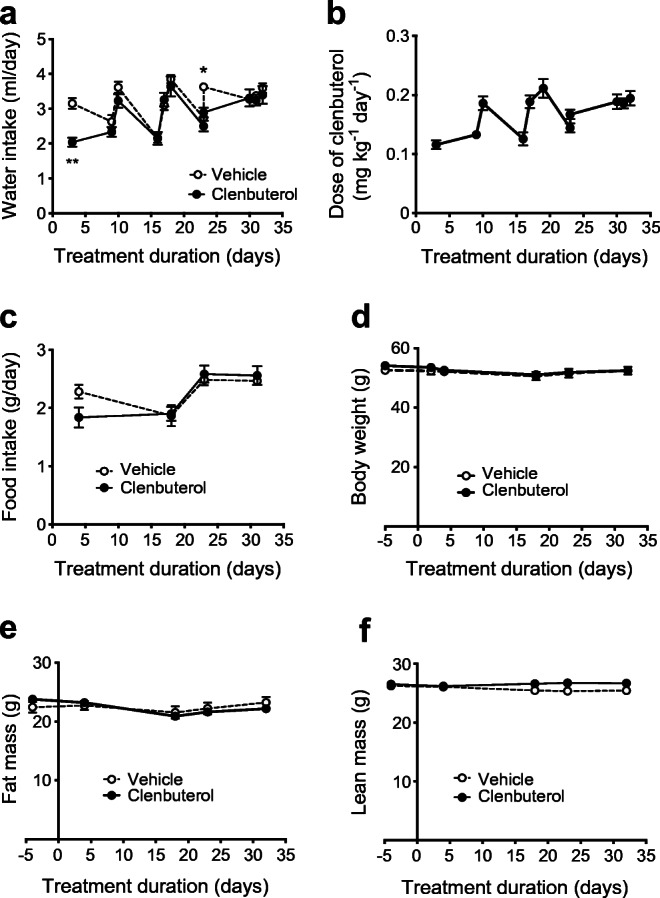
Effects of prolonged treatment with a low dose of clenbuterol on food and water intake, body weight and body composition. DIO mice were treated with a low dose of clenbuterol for 32 days; *n* = 6–7. Diet-induced obesity was developed in C57Bl/6N mice maintained at 30°C and on HFD for 5 months; 3 mg/l clenbuterol was administered in drinking water. (**a**) water intake, (**b**) dose of clenbuterol calculated based on water intake, (**c**) food intake, (**d**) body weight, (**e**) body fat mass, (**f**) body lean mass. In (**a**) the data were analysed by mixed-effects analysis (since a few random data points were missing owing to water bottle leakage) with Sidak’s multiple comparison test; in (**c–f**) the data were analysed by two-way ANOVA with Sidak’s multiple comparison test; in all graphs the experimental group was not significantly different from the vehicle group

When evaluated by OGTT, glucose tolerance improved after 4 days of low-dose clenbuterol treatment (ESM Fig. 2a) and this effect persisted when the treatment was continued for a prolonged period of time (ESM Fig. 2b), similar to IPGTT in Fig. 2c–f. Treatment with clenbuterol did not significantly affect either water or food intake (Fig. 4a, c). Importantly, body weight, fat mass and lean mass remained unaffected during the treatment period (Fig. 4d–f).

**Fig. 5:**
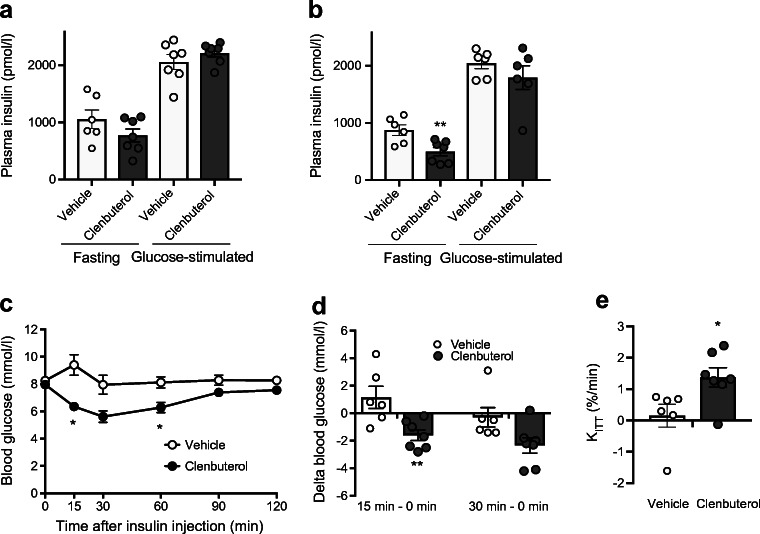
Prolonged treatment with a low dose of clenbuterol does not increase insulin levels (OGTT) but improves insulin sensitivity. The data are from the same experiment as in Fig. 4 and ESM Fig. 2. Mice were fasted for 5 h. Plasma insulin was measured before and 15 min after glucose gavage in mice pre-treated with clenbuterol for 4 (**a**) and 18 (**b**) days; *n* = 6–7. The data were analysed with unpaired two-tailed Student’s *t* test separately for fasting and glucose stimulated conditions. (**c**) ITT performed on 25th day of treatment with clenbuterol. After 5 h of fasting, insulin (1 U/kg body weight) was injected i.p. and blood glucose was measured after 15, 30, 60, 90 and 120 min; *n* = 6–7. Insulin response from (**c**) is presented as change of blood glucose (15 min minus 0 min) and (30 min minus 0 min, *p* = 0.056) (**d**), and rate of blood glucose disposal (*K*_ITT_) (**e**). The data in (**c**, **d**) were analysed by two-way ANOVA with Sidak’s multiple comparison test, and in (**e**) with unpaired two-tailed Student’s *t* test. In all graphs: *p < 0.05, **p < 0.01 vs vehicle

Fasting insulin levels were unchanged after 4 days of clenbuterol treatment (Fig. 5a), whereas they were significantly lowered by 43% after 18 days of treatment (*p* < 0.01, Fig. 5b). Fifteen minutes after glucose oral gavage, plasma insulin levels increased to the same extent in control and clenbuterol-treated mice both at 4 and 18 days of treatment (Fig. 5a, b), indicating that improvements in glucose tolerance were not a consequence of higher insulin release.

Although clenbuterol administration did not result in a higher insulin release during the OGTTs, it could still potentially improve glucose tolerance by increasing insulin sensitivity. To explore this possibility, we performed an ITT after 24 days of low-dose clenbuterol treatment. As expected, blood glucose of DIO mice in the control group did not respond to insulin injection, indicating insulin resistance. Remarkably, clenbuterol-treated DIO mice showed a significant improvement of insulin sensitivity**.** Rates of glucose disposal (*K*_ITT_) were markedly increased from 0.15 ± 0.36 to 1.38 ± 0.31%/min in control vs treated groups, respectively, *p* < 0.05 (Fig. 5c–e). In fact, clenbuterol fully abolished insulin resistance caused by high-fat diet, since clenbuterol-treated mice responded to insulin similarly to chow-fed mice, which were, however, tested at a separate occasion (ESM Fig. 3).

Together, these data indicate that the beneficial glucose-lowering effect of clenbuterol manifested on glucose tolerance is not due to increased insulin secretion but involves increases in insulin sensitivity.

### Adaptations in liver and skeletal muscle underlie the clenbuterol-mediated improvements in glucose homeostasis

**Fig. 6:**
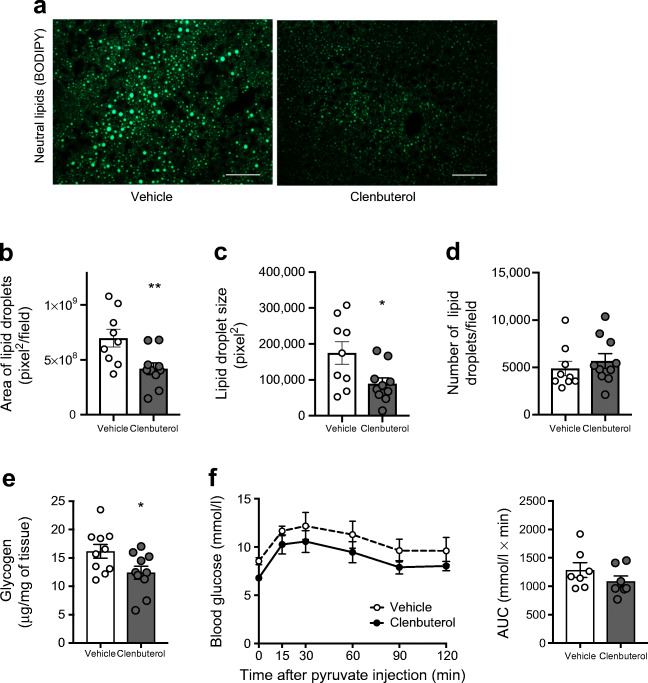
A low dose of clenbuterol reduces hepatic steatosis and hepatic glycogen. (**a–e**) DIO mice were treated with clenbuterol for 32 days; *n* = 9–10, both males and females were used. Diet-induced obesity was developed in C57Bl/6N mice maintained at 30°C and on HFD for 7 months; 3 mg/l clenbuterol was administered in drinking water; mean actual dose of clenbuterol calculated based on water intake was 0.16 mg kg^−1^ day^−1^; mice were fasted for 5 h in the morning before dissections. (**a**) Representative fluorescent images of liver lipid droplets stained with BODIPY (green, lipid); scale bars, 50 μm. Quantification of images in (**a**) using ImageJ, in terms of total area of lipid droplets (**b**), size (**c**) and number of lipid droplets (**d**). (**e**) Liver glycogen. (**f**) Pyruvate tolerance test was performed after 9 days of treatment during the same experiment as in Figs 4, 5 and ESM Fig. 2. The data were analysed with unpaired two-tailed Student’s *t* test in (**b**–**e**) and AUC in (**f**). In all graphs: *p < 0.05, **p < 0.01 vs vehicle

To further elaborate on the mechanisms that could underlie the improvements in glucose tolerance and insulin sensitivity upon clenbuterol treatment, we focused on the liver and skeletal muscle. As hepatic lipid accumulation is closely associated with insulin resistance [24–27], we first assessed hepatic lipid content by histological analysis. The area of hepatic lipid droplets was markedly reduced by ~40% (*p* < 0.01) after prolonged (32 days) low-dose clenbuterol treatment. This reduction in hepatic steatosis was entirely due to a decrease in lipid droplet size (49%, *p* < 0.05), since number of lipid droplets remained unaffected (Fig. 6a–d). A gravimetric assay for total amount of extracted lipids generated similar results (ESM Fig. 4). Apart from lipids, prolonged clenbuterol treatment decreased liver glycogen by 23% (*p* < 0.05, Fig. 6e) but did not affect gluconeogenesis, as addressed by pyruvate tolerance test (Fig. 6f).

**Fig. 7:**
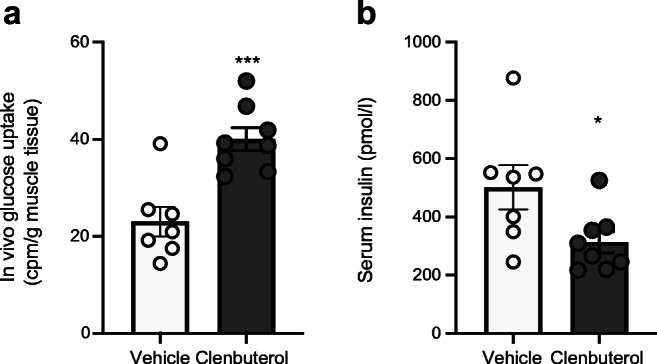
A low dose of clenbuterol stimulates in vivo glucose uptake in gastrocnemius muscle of DIO mice during chronic treatment. (**a**, **b**) DIO was developed in C57Bl/6N mice maintained at 30°C and on HFD for 8 months. DIO mice were treated with saline (vehicle; *n* = 7) or clenbuterol (*n* = 8) for 6 days. After 4 days of treatment, IPGTT was performed (not shown). After 6 days of treatment in vivo glucose uptake was measured in gastrocnemius muscle, 5 h after the last treatment and after 5 h of fasting (**a**). Insulin was measured in serum collected during the test (**b**). The data were analysed with unpaired two-tailed Student’s *t* test: *p < 0.05, ***p < 0.001 vs vehicle

To test whether basal glucose uptake in skeletal muscle was stimulated upon chronic low-dose clenbuterol treatment in DIO mice, we performed in vivo glucose uptake assay after 6 days of treatment. Low doses of clenbuterol stimulated glucose uptake in skeletal muscle by 74% even 5 h after the last treatment (*p* < 0.001, Fig. 7a). Importantly, stimulated glucose uptake was not attributed to increased insulin levels. In fact, serum insulin was reduced in clenbuterol-treated mice during the experiment (by 38%, *p* < 0.05, Fig. 7b, similarly to Fig. 5b).

Combined, these data suggest that adaptations in both the liver and skeletal muscle as well as an increase in insulin sensitivity contribute to the clenbuterol-mediated improvements in glucose homeostasis.

## Discussion

In the present study, we investigated the therapeutic relevance of β_2_-AR stimulation on glucose homeostasis in DIO mice. We demonstrated that treatment with the β_2_-adrenergic agonist clenbuterol improves glucose tolerance already after 4 days of treatment. These effects persisted at least up to 42 days of treatment and could also be achieved with doses in the microgram range, which are about 40 times lower than previously studied [2, 4, 15, 28]. Moreover, low-dose clenbuterol stimulated basal in vivo glucose uptake in skeletal muscle and improved whole-body insulin sensitivity as well as reduced hepatic steatosis under chronic stimulation in DIO mice.

**Fig. 8:**
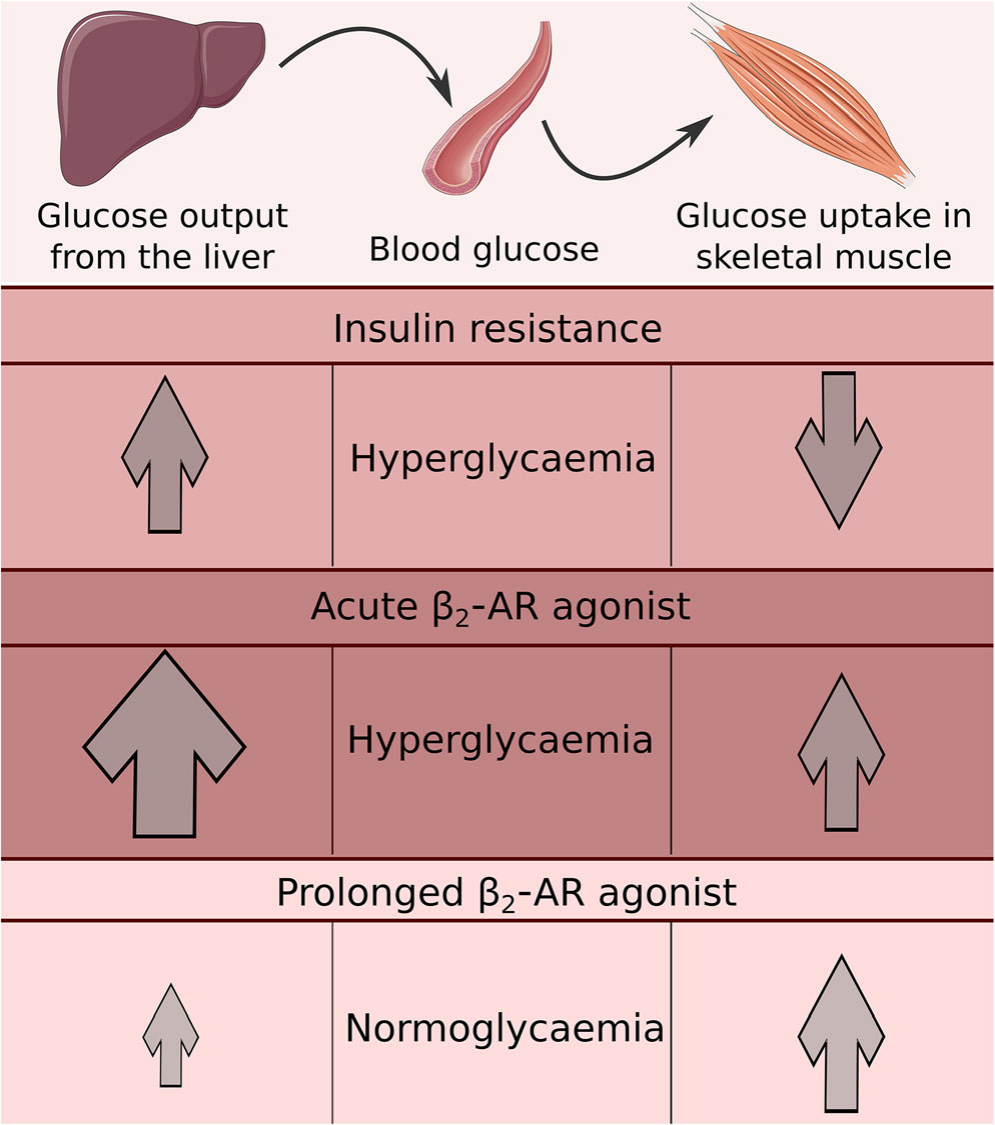
Working hypothesis of clenbuterol actions acutely and chronically. Prediabetic and diabetic people and rodents are characterised by insulin resistance, which leads to increased glucose output from the liver and reduced glucose uptake in peripheral tissues, including muscle, and which results in hyperglycaemia and glucose intolerance. Treatment with a β_2_-AR agonist such as clenbuterol (acutely or chronically) stimulates glucose uptake in skeletal muscles. However, acutely clenbuterol augments hepatic glucose output, which is not fully compensated by enhanced glucose uptake in peripheral tissues and results in hyperglycaemia as a net effect. However, under prolonged treatment with clenbuterol, peripheral glucose clearance matches hepatic glucose output, resulting in a favourable metabolic profile. Hepatic glucose output is likely to be reduced under prolonged clenbuterol treatment at least in part because of normalised insulin sensitivity and reduced liver glycogen. This working hypothesis is elaborated in the discussion section. Images were taken from Servier Medical Art (smart.servier.com)

In the present study, and in line with previous findings, acute clenbuterol administration increased blood glucose and insulin levels [7–10]. However, we could also show that, after 4 days of treatment, clenbuterol reduced fasting blood glucose and improved glucose tolerance (24 h after last administration), and these effects were maintained at least up to 42 days. On the basis of these combined results we hypothesised that treatment with β_2_-adrenergic agonists induces a dynamic response over time (Fig. 8). Thus, clenbuterol (acutely or chronically) stimulates glucose uptake in skeletal muscles. However, in the acute setting, clenbuterol augments hepatic glucose output, which is not fully compensated by the enhanced glucose uptake in peripheral tissues resulting in hyperglycaemia. Upon prolonged treatment with clenbuterol, peripheral glucose clearance appears to exceed the hepatic glucose output, resulting in a favourable metabolic profile. Hepatic glucose output is likely to be diminished under prolonged clenbuterol treatment at least in part due to improved insulin sensitivity and reduced glycogen levels.

Regarding the underlying mechanism, we first investigated how a low dose of clenbuterol affects body composition. Clenbuterol is well known for its capacity to increase lean mass and decrease fat mass [29, 30]. In turn, improvements in glucose homeostasis could be attributed to any of these two effects [31, 32]. However, clenbuterol did not affect either lean or fat mass in the current study, presumably owing to the low dose of clenbuterol applied. We also excluded enhanced insulin secretion as a contributing factor since fasting plasma insulin concentrations were significantly reduced upon prolonged low-dose clenbuterol treatment and since plasma insulin levels rose to a similar extent in treated vs non-treated mice upon glucose administration during OGTTs.

In the absence of changes in insulin secretion, we hypothesised that the improvements in glucose tolerance were linked to an increased insulin sensitivity. Indeed, whole-body insulin sensitivity improved remarkably upon prolonged low-dose clenbuterol treatment, as assessed by ITT. These results confirm previously published data, which, however, were obtained with a relatively high dose of clenbuterol [15]. It is a remarkable effect considering that insulin resistance is a characteristic manifestation of type 2 diabetes, it is closely associated with several other conditions including cardiovascular diseases and it remains poorly treated to date [33]. Here we show that selective β_2_-adrenergic agonists present an attractive avenue for treatment of insulin resistance.

The underlying mechanism by which β_2_-adrenergic agonists beneficially affect insulin sensitivity remains to be elucidated. Although these effects have previously been related to tissue redistribution [4, 15], we excluded this factor as was discussed above. Instead, the insulin-sensitising effects of clenbuterol could be, in part, mediated by reduction in hepatic lipid accumulation [34–36]. Remarkably, we could show that total liver lipid content was drastically reduced upon clenbuterol treatment. These results contradict previous data on isoprenaline (isoproterenol), which stimulated hepatic lipid accumulation in mice [37]; the difference however may be accounted for by the fact that isoprenaline is a non-selective β-AR agonist, which moreover was administered at a very high dose. Another possible mechanism for how clenbuterol could improve insulin sensitivity, particularly in the liver, is by reducing liver glycogen levels. An experimentally-increased amount of glycogen in the liver by itself induced hepatic insulin resistance and inhibited new glycogen synthesis in healthy dogs [38]. Interestingly, novel classes of glucose-lowering drugs, SGLT2 inhibitors and dual GLP1/glucagon agonists, are also known to reduce liver glycogen storage [39–41]. In addition, clenbuterol could also affect insulin sensitivity through a reduction in inflammation, since these two conditions are closely associated (reviewed in [42, 43]). In fact, other β_2_-adrenergic agonists, salbutamol and terbutaline, exhibited anti-inflammatory effects in diabetic rats and DIO mice [44, 45]. Ultimately, corrected hyperglycaemia can also improve insulin sensitivity, since glucotoxicity by itself can lead to insulin resistance in healthy men and mice [46, 47].

Most importantly, one consequence of improved insulin sensitivity is reduced (better controlled) hepatic glucose output, which in turn is an important contributor to normal glucose homeostasis (see working hypothesis in Fig. 8). Moreover, lower hepatic glycogen levels may reduce glucose output from the liver, since the rate of glycogenolysis is known to depend on the amount of glycogen [48]. Collectively, higher insulin sensitivity and lower glycogen in the liver of clenbuterol-treated mice may contribute to decreased (normalised) glucose output from the liver and, together with stimulated glucose disposal in muscles, lead to restored glucose homeostasis (Fig. 8).

Besides the liver, we also studied skeletal muscle for its contribution to the clenbuterol-induced improvements in glucose homeostasis. We clearly showed that chronic treatment with clenbuterol stimulated basal glucose uptake in skeletal muscle in vivo independently of insulin*,* indicating an intrinsic ability of adrenergic stimulation to induce glucose uptake in skeletal muscles in vivo (probably through activation of the β_2_-mTORC2 pathway [2]). Interestingly, stimulation of basal glucose uptake in muscles was able to improve glucose homeostasis in mice overexpressing GLUT1 in skeletal muscle [49]. Thus, our findings support that prolonged treatment with the β_2_-adrenergic agonist clenbuterol stimulates basal glucose uptake in skeletal muscle, which, together with the previously established effect on insulin-stimulated glucose uptake, is likely to contribute to the observed improvements in whole-body glucose homeostasis.

Human relevance of the β_2_-adrenergic pathway was indicated in our previous study, in which mild cold exposure stimulated glucose uptake in skeletal muscle and improved insulin sensitivity in diabetic people without activation of insulin- or AMPK-signalling [50]. In another study on healthy men, insulin sensitivity was improved under chronic treatment with β_2_-AR agonist terbutaline (15 mg/day for 1–2 weeks) [18]. In the present study we demonstrated that clenbuterol improved glucose tolerance in DIO mice at doses in the microgram range, which are generally assumed safe for treatment of asthma [20–22]. Demonstration of effectiveness of clenbuterol at much lower doses than was previously studied is important, since reducing the dose of the drug may diminish potential side effects. As such, the true clinical relevance of this novel, insulin-independent pathway to improve whole-body glucose homeostasis (namely, three conditions: glucose tolerance, insulin resistance and hepatic steatosis) could be readily tested in humans.

Our study has its limitations. DIO mice are characterised by insulin resistance but only mild hyperglycaemia. Thus, they can be interpreted as a model for only early stages of type 2 diabetes. However, clenbuterol has been shown to improve glucose homeostasis in two other models [2, 4, 15]. Second, it would be of clinical relevance to extend our study to other β_2_-AR agonists, which are currently in wider clinical use for asthma. To date there are only few studies addressing these objectives [16–18]. Third, our study was almost entirely limited to male mice, partly due to relative resistance of female C57Bl/6 mice to developing obesity, glucose intolerance and insulin resistance.

In summary, prolonged supplementation of a β_2_-AR agonist robustly improved glucose homeostasis in DIO mice already after 4 days of treatment. Remarkably, these effects could be maintained for up to 42 days and could be achieved with very low doses. These effects on glucose homeostasis were most likely to be mediated both by an enhanced glucose uptake in skeletal muscle and an improved whole-body insulin sensitivity. In addition, prolonged treatment with clenbuterol reduced hepatic glycogen and profoundly improved hepatic steatosis.

## Electronic supplementary material

ESM 1 (PDF 283 kb)

## Data Availability

The datasets generated during the current study are available from the corresponding author on reasonable request.

## Abbreviations

AMPK: AMP-activated protein kinase
AR: Adrenergic receptor(s)
DIO mice: Mice with diet-induced obesity
HFD: High-fat diet
mTORC2: Mammalian target of rapamycin complex 2
