# Supplementary material for: Treatment with a β-2-adrenoceptor agonist stimulates glucose uptake in skeletal muscle and improves glucose homeostasis, insulin resistance and hepatic steatosis in mice with diet-induced obesity
Source: Diabetologia. 2020 May 29;63(8):1603–15. doi: 10.1007/s00125-020-05171-y (PMC7351816; doi:10.1007/s00125-020-05171-y)
Supplement: Supplementary file 1 — (PDF 283 kb) [file 125_2020_5171_MOESM1_ESM.pdf]

ELECTRONIC SUPPLEMENTARY MATERIAL (ESM)

METHODS (detailed version)

Animals The animals for these experiments were male C57Bl/6N mice, bred in-house at Stockholm University or purchased in Scanbur (Charles River). To generate diet-induced obesity, mice were fed a high fat and high sucrose diet (HFD) (45% fat kcal from fat and 17% kcal from sucrose, D12451; Research Diets, New Brunswick, NJ) *ad libitum* and kept in a thermoneutral (30_0 C temperature) room with a 12-h light/dark cycle. Depending on the experiment, mice were routinely fed HFD for 4-9 months prior to start of the treatment as well as during the treatment until the end of the experiment. Mean body weight of DIO mice was 40-50 g. DIO mice were males unless stated otherwise and 7-10 months old in the beginning of the treatment. Mice had unlimited access to water. Mice were housed in individual cages during the treatment, unless stated otherwise. For the acute *in vivo* glucose uptake experiment in healthy mice, 10 weeks old male, chow-fed mice were used, which were kept grouped caged at 21°C; their body weight was around 25 g. Animal experiments were performed in specific pathogen free animal house. Cages were enriched with wood chips, a cardbord house or a roll, a wooden stick as well as paper and a piece of cotton as nesting material. Body composition was measured by an *in vivo* magnetic resonance imaging technique using EchoMRI-100™ (Echo Medical Systems, USA). All procedures involving animals were approved by the North Stockholm Ethical Committee for Care and Use of Laboratory Animals**.**

Treatment Prior to treatment mice in different experimental groups had similar glucose tolerance, body weight and body composition. The β_2-adrenergic agonist clenbuterol (C5423, Sigma Aldrich, ≥ 95 %) was injected i.p. (dissolved in saline [154 mmol/l NaCl]) or supplied in the drinking water as indicated in the figure legends. In experiments, where clenbuterol was supplied in drinking water, water was changed every 2-3 days. In chronic experiments, where clenbuterol was administered with i.p. injections, treatment was performed daily in the mornings. Control and treated mice were treated in random order.

GTT, ITT and pyruvate tolerance test For the glucose tolerance tests, mice were fasted for 5 hours in the beginning of light phase. An intraperitoneal glucose tolerance test (IPGTT) or oral glucose tolerance test (OGTT) was performed around 12-13 pm. Glucose 2.5 g per kg lean

weight unless stated otherwise was dissolved in saline and either injected i.p. or orally gavaged. Blood glucose was measured at baseline (time point 0) as well as 15, 30, 60, 90 and 120 minutes after the glucose challenge, in blood collected from the tail vein using an Accu-chek Aviva (Roche Diagnostics Scandinavia AB, Stockholm, Sweden). The AUC for glucose tolerance was calculated from 0 to 120 min as the geometrical area with 0 mmol/l of blood glucose as a baseline. Please note, that effect on fasting blood glucose contributes to the effect on total AUC. During OGTTs, plasma samples were collected for assessment of insulin concentrations at time points 0 and 15 minutes after glucose challenge. For this, 20-30 µl of blood was collected in capillary tubes supplemented with EDTA (16.444.100, Sarstedt, Helsingborg, Sweden) from a cut in the tail tip and centrifuged at 3000 g for 10 minutes. Subsequently, plasma was collected and kept at -80°C until insulin measurement. Plasma insulin was measured by means of Ultra Sensitive Mouse Insulin ELISA Kit (90080, Crystal Chem, Zaandam, Netherlands).

For ITT mice were fasted for 5 hours in the beginning of light phase, after which human insulin at a dose of 1 U/kg body weight (Actrapid, Novo Nordisk, Copenhagen, Denmark) dissolved in saline was injected intraperitoneally. Blood glucose was measured as described above at t = 0, 15, 30, 60, 90 and 120 min after the insulin injection. Rate of blood glucose disposal (K_{ITT}) was calculated as $0.693\times100/t_{1/2}$, where $t_{1/2}$ is the time necessary to reduce blood glucose by half, calculated from the linear regression analysis of data at t = 0, 15 and 30 minutes [1]. Delta blood glucose – 15 min *minus* 0 min and 30 min *minus* 0 min – were also calculated.

For the pyruvate tolerance test mice were fasted for 12 hours during light phase (7 am – 7 pm) at 21°C in order to empty liver glycogen storage as much as possible and thus reduce contribution of glycogenolysis. Two hours prior to the test mice were moved back to 30°C, where the test was performed. 2.5 g of pyruvate per kg lean weight (P8574, Sigma Aldrich) dissolved in saline was injected i.p. Blood glucose was measured as described above for GTTs at 0, 15, 30, 60, 90 and 120 min time points. The AUC for pyruvate tolerance was calculated from 0 to 120 min as the geometrical area with 0 mmol/l of blood glucose as a baseline.

During GTTs, ITT and pyruvate tolerance test mice were kept in their home cages in the 30°C room. Clenbuterol was not administrated at the day of a test. Control and treated mice were accessed in random order.

***In vivo* glucose uptake** Acute clenbuterol treatment in lean mice (Fig. 1D): male C57Bl/6N mice purchased from Scanbur (Charles River) were used. Mice were 10 weeks old, fed a chow diet and kept group caged at 21°C. Mice in two experimental groups had similar body weight, body fat and lean mass. Mice were fasted for 5 hours in the beginning of light phase, around 12-13 pm

anesthetized with pentobarbital (60 mg/kg i.p.), placed on a 37 °C pad and injected intraperitoneally with clenbuterol (1 mg/kg) or saline. Clenbuterol did not affect blood glucose of anaesthetized mice when measured 20 minutes after clenbuterol injection (not shown); thus, glucose uptake was not influenced by different blood glucose levels. 20 min after saline/clenbuterol injection, $4.81 \cdot 10^6$ Bq/kg of 2-deoxy[^{3}H]glucose (Perkin Elmer, Waltham MA USA; $2.96 \cdot 10^{11}$ Bq/mmol) was injected intraperitoneally. Animals were euthanized 1 hour later by CO_2.

Prolonged clenbuterol treatment in DIO mice (Fig. 7A, B): diet-induced obesity was developed in C57Bl/6N mice purchased from Scanbur (Charles River) and maintained at 30°C and on HFD for 8 months, mice were 11 months old in the end of the experiment. 0.17 mg/kg clenbuterol was injected i.p. daily. After 4 days of treatment an ipGTT was performed (not shown). On the 7th day of treatment *in vivo* glucose uptake was performed 5 hours after the last treatment with saline/clenbuterol: mice were fasted for 5 hours in the beginning of light phase, around 12-13 pm anaesthetized with pentobarbital (70 mg/kg i.p.), placed on a 37°C pad and injected with $4.81 \cdot 10^6$ Bq/kg of 2-deoxy[^{3}H]glucose (Perkin Elmer, Waltham MA USA; $2.96 \cdot 10^{11}$ Bq/mmol, i.p.). 80 minutes later mice were euthanized by CO_2. Isotope enrichment was measured at t=20 min after 2-deoxy[^{3}H]glucose injection. Normalization of glucose uptake to isotope enrichment yielded similar results (not shown). 20 min after 2-deoxy[^{3}H]glucose injection serum was collected and insulin was measured as described above.

In both experiments, the heart was perfused with 20 ml of cold phosphate-buffered saline. Gastrocnemius muscle was dissected out and lysed in 0.5 M NaOH. Tissue lysate was mixed with scintillation buffer 1:20 (Emulsifier Safe, Perkin Elmer) and radioactivity was detected in a beta-counter (Tri-Carb 4810TR, Perkin Elmer, USA). Each data point is from a single animal. Control and treated mice were accessed in mixed order. In all stages investigators were blinded to treatment.

Hepatic lipids BODIPY staining of lipid droplets: DIO mice were fasted for 5 hours in the beginning of the light phase (7 am -12 pm), euthanized by CO_2 and their hearts were perfused with 10 ml of cold phosphate-buffered saline. Piece of liver was immediately frozen in melting isopentane cooled with liquid nitrogen. Subsequently, tissues were kept at -80°C, until the further analysis. The lipophilic fluorescence dye BODIPY 495/503 (D3922, Molecular Probes, Carlsbad, Calif, USA) was used to visualize lipid droplets in frozen liver sections. BODIPY was diluted in phosphate-buffered saline at a concentration of 0.1 mg/mL and applied to liver sections for 90 minutes at 37 °C; 4,6-diamidino-2-phenylindole (DAPI) was used to identify nuclei. All

samples were mounted in Mowiol and covered with #1 glass coverslips. Digital images were obtained using a E800-fluorescence microscope (Nikon) and were analyzed for lipid droplet size and number with ImageJ.

Lipids amount: mice were acclimatized either to 21°C or 30°C, have been on a high-fat diet for 4 months prior to treatment and treated with clenbuterol (3 mg/l in drinking water) for 40 days. Acclimatization to different temperatures in this experiment did not affect either body weight or glucose tolerance or amount of hepatic lipids. Mice were not fasted, euthanized by CO_2 and their hearts were perfused with 20 ml of cold phosphate-buffered saline. Piece of liver was immediately frozen in liquid nitrogen and then kept at -80°C until the further analysis. Piece of liver (around 100-200 mg) was placed in pre-weighed glass vials and carefully weighed. 5 ml of methanol:chloroform extraction medium (1:2, vol./vol.) was added to the tissue and kept at room temperature for 2 days, after which the extract was transferred to another pre-weight vial and further 2 ml of the same extraction medium was added to the original vial. 2 days later second extract was added to the first one. Extraction medium was evaporated and lipids weight was determined gravimetrically.

Hepatic glycogen DIO mice were fasted for 5 hours in the beginning of the light phase (7 am -12 pm) and euthanized by CO_2, hearts were perfused with 10 ml of cold phosphate-buffered saline. A piece of liver was dissected, immediately frozen in liquid nitrogen and then kept at -80 °C until further analysis. 10 mg of each tissue sample were resuspended on ice in 0.4 ml of water and homogenized with an ultra-turrax mixer for 20 seconds. Homogenates were boiled for 10 minutes to inactivate enzymes and centrifuged for 10 minutes at 18,000*g* at 4 °C to remove any insoluble material. 2 µl of supernatant was assayed in duplicates using a kit (ab65620, Abcam, UK) and protocol provided by manufacturer.

Cell culture L6 rat skeletal muscle myoblasts as well as L6 skeletal muscle cells that stably express GLUT4-myc were purchased from KeraFast (ESK201 and ESK202, respectively; Boston, MA, USA), where they were tested for mycoplasma. Normal morphology and growth were confirmed in all cell cultures used in the experiments. Cells were kept in low glucose (1 g/L) Dulbecco's Modified Eagle Medium (DMEM) supplemented with 10% fetal bovine serum, 10 mmol/l HEPES, 4 mmol/l L-glutamine and 100 units/ml penicillin and 100µg/ml streptomycin (all from Sigma Aldrich) in a 37 °C incubator with 5% CO_2. After seeding cells, the cells were growing 90% to confluence and then exposed to differentiation media, containing 2% fetal bovine serum until formation of myotubes, i-e. 5-7 days.

In vitro glucose uptake After differentiation, L6 cells were serum starved for 3.5 hours in low glucose DMEM supplemented with 0.5 % bovine serum albumin (33046827, Roche, Mannheim, Germany). Clenbuterol in different doses (C5423, Sigma Aldrich) dissolved in water was added for stimulation for 1.5 hours. Cells were then carefully washed with 37 °C glucose free media and clenbuterol/saline was re-added. After incubation for 20 min, cells were then exposed to 50 nmol/l 2-deoxy[3H]glucose (Perkin Elmer, Waltham MA USA) for another 10 min, afterwards they were washed in ice-cold glucose free medium and lysed with prewarmed (60° C) 0.2 M NaOH. Cell lysates were mixed with scintillation buffer 1:10 (Emulsifier Safe, Perkin Elmer) and radioactivity was detected in a beta-counter (Tri-Carb 4810TR, Perkin Elmer). Each data point is a mean value of 2 wells-duplicates from a separate experiment.

In vitro GLUT4 translocation L6 skeletal muscle cells that stably express GLUT4-myc were seeded on 2% gelatine coated object glasses with a concentration of 100000 cells/ml. After confluence, the cells were differentiated until formation of myotubes. The cells were then serum starved for 3 hours and stimulated for 2 hours with 1 µM clenbuterol or corresponding control. After stimulation, the cells were fixed with 2% 37°C paraformaldehyde for 15 min and washed quickly with phosphate-buffered saline. Before incubation with primary antibody (rabbit anti-myc, 2278 from Cell Signaling, diluted 1:500 in PBS with 5% BSA) overnight in 4 °C, the glasses were quenched and blocked for 1 hour in room temperature with 50 mmol/l glycine and 5% bovine serum albumin diluted in phosphate-buffered saline. The second day, the glasses were incubated in the dark for 1 hour with conjugated Alexa Fluor555 goat anti-rabbit antibody (21429 from Invitrogen, diluted 1:500 in PBS with 1.5% BSA). Dapi staining was in the mounting media (Vectashield) and coverslips were sealed with nail polish. Fluorescence was detected with a fluorescent confocal microscope (Zeiss LSM 800). Three cells from each of three separate experiments were randomly picked and analysed by the light intensity with Image J. Each data point is a mean of the 3 cells. When myc-epitope was probed on the cells by Western blot, it resulted in only one band of a right molecular weight (not shown). Omission of the primary antibody resulted in no staining of the cells, confirming specificity of the secondary antibody.

Statistical Analysis All data are expressed as the mean ± SEM. In animal experiments each data unit is a single mouse. In in vitro experiments (Fig. 1 A-C) each data point was derived from a separate experiment and is a mean of two duplicates (fig 1A) or three triplicates (fig 1C).

Criteria for data exclusions were: obvious pipetting errors using insulin Elisa kits, which resulted in almost no signal (one value from each control and glucose groups on Fig. 1f; one control value on Fig. 5a and one treated value on Fig. 5b); not properly injected glucose during IPGTT not resulted in rise of blood glucose (two control values on Fig. 3a,b); water leakage resulted in too high apparent water intake (few days in all groups on Fig. 4a).

Data were analyzed with unpaired two-tailed Student's T-test, one-way or two-ways ANOVA with the Dunnett's or Sidak's multiple comparison tests or with mixed-effect analysis as indicated in figure legends. Statistical analyses were performed using GraphPad Prism software 8.2 (GraphPad Software, Inc. La Jolla, CA, USA). A significant difference was considered at * $p < 0.05$, ** $p < 0.01$, *** $p < 0.001$.

1. Bonora E, Moghetti P, Zancanaro C, et al (2009) Estimates of in vivo insulin action in man: comparison of insulin tolerance tests with euglycemic and hyperglycemic glucose clamp studies. J Clin Endocrinol Metab 68(2):374–378. https://doi.org/10.1210/jcem-68-2-374

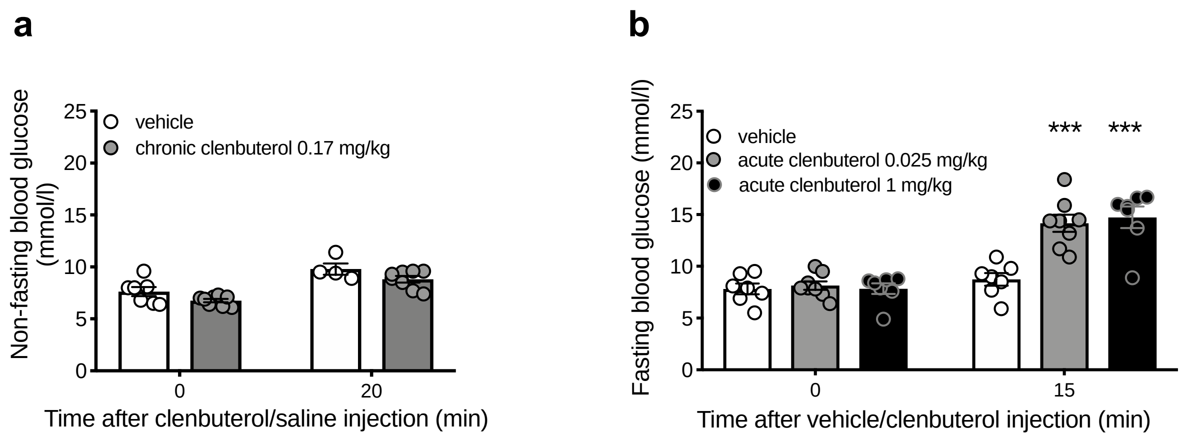

ESM Fig. 1. Repetitive daily administration of clenbuterol did not increase blood glucose.

(a) DIO was developed in C57Bl/6N mice maintained at 30°C and on HFD for 8 months. DIO mice were injected i.p. with saline (n=7) or clenbuterol (0.17 mg kg^{-1} day^{-1}, n=8) for 7 days. On the last day of the treatment, non-fasting blood glucose was measured before and 20 min after drug administration. Data were analyzed by mixed-effects analysis with Dunnett's multiple comparison test. **(b)** acutely, single injection with clenbuterol elevates fasting blood glucose even at the lowest dose of the drug (0.025 mg/kg). Data were analyzed by two-way ANOVA with Dunnett's multiple comparison test. *** $p < 0.001$.

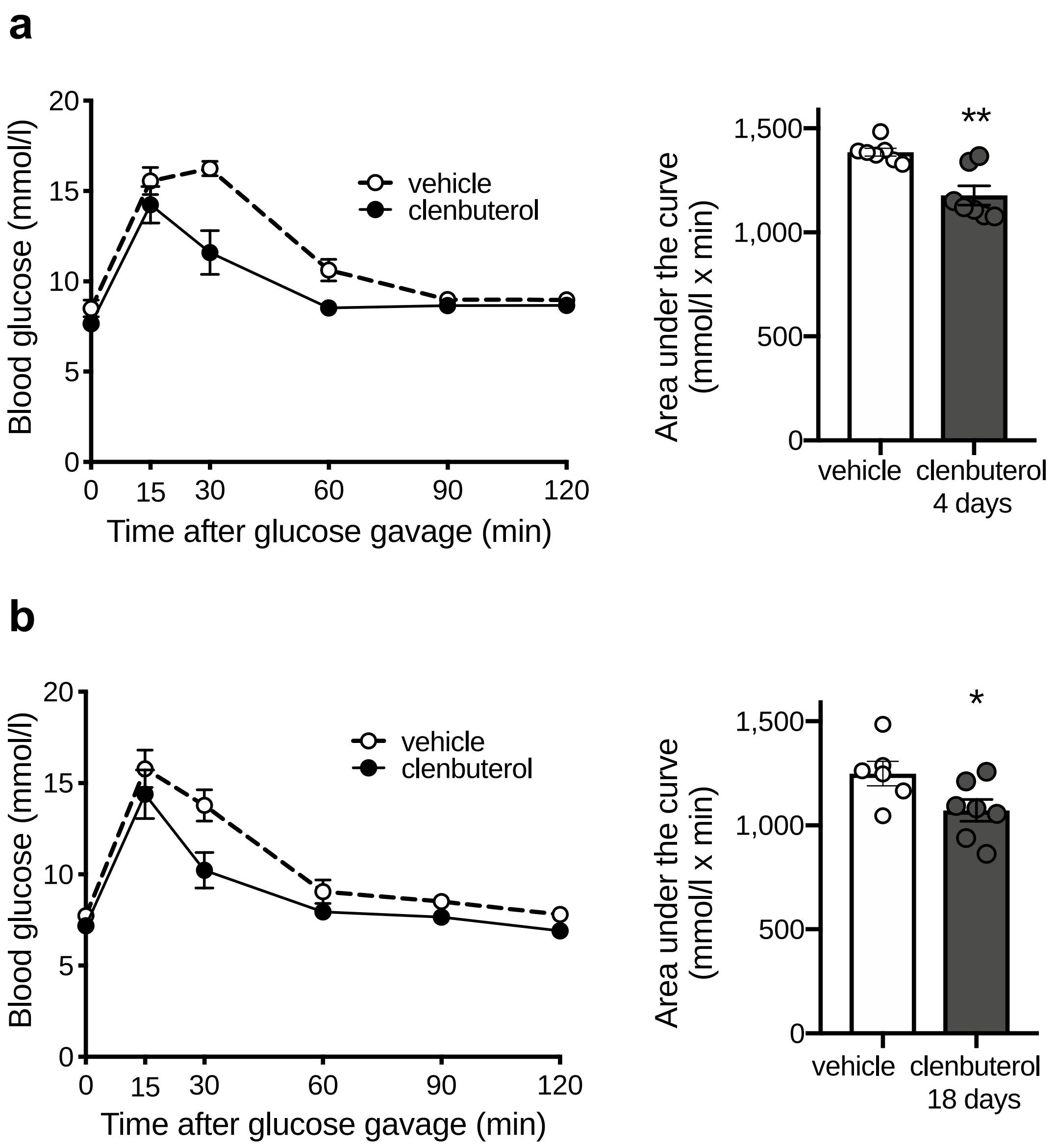

ESM Fig. 2. Prolonged treatment with low-dose clenbuterol improves oral glucose tolerance.

The data are from the same experiment as in figures 4, 5 and 6f. DIO mice were treated with clenbuterol (3mg/l in drinking water) for 32 days. Oral GTT was performed after 4 **(A)** and 18 **(B)** days of the treatment. After 5 hours of fasting, glucose (2,5 mg/kg lean mass) was administrated by oral gavage and blood glucose was measured in 15, 30, 60, 90 and 120 minutes. AUC were analyzed with unpaired two-tailed Student's T-test. * $p < 0.05$, ** $p < 0.01$.

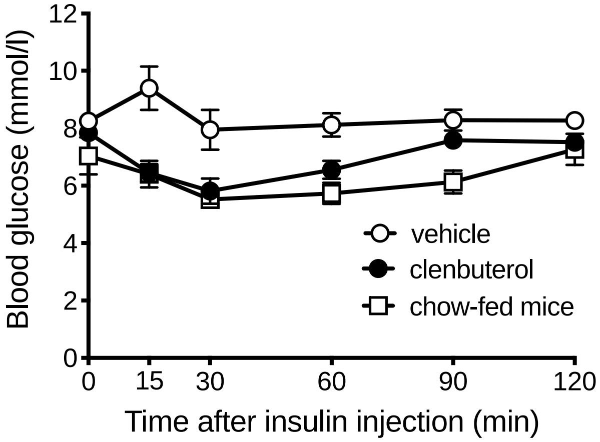

ESM Fig. 3. Prolonged treatment with low dose of clenbuterol normalizes insulin sensitivity to the level of young chow-fed mice kept at room temperature.

Data for vehicle and clenbuterol are the same as in figure 5C. Please note, that chow-fed mice are from a separate experiment, performed at a different occasion (mice were not treated). Data in clenbuterol and chow-fed groups were not statistically different at time points 0, 15 and 30 minutes, as analyzed by two-way ANOVA with Tukey's multiple comparison test.

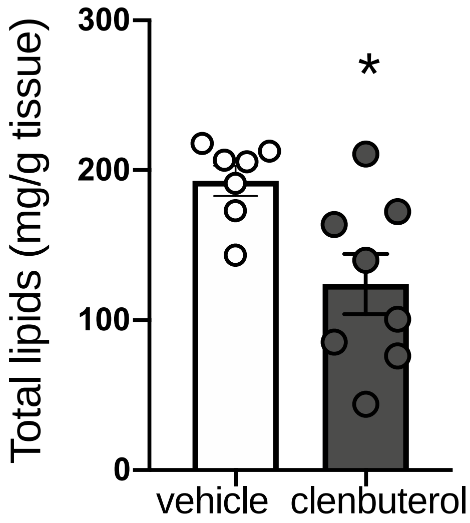

ESM Fig. 4. Prolonged treatment with low dose of clenbuterol reduces hepatic steatosis. Diet-induced obesity was developed in C57Bl/6N mice maintained at 21°C or 30°C and on HFD for 4 months; they were treated with clenbuterol for 40 days, 3 mg/l clenbuterol was administered in the drinking water. Mice were 10 months old in the beginning of the treatment. Hepatic lipids were extracted and measured gravimetrically. Data were analyzed by unpaired two-tailed Student's T-test. * $p < 0.05$.
